# Head to head comparison of diagnostic performance of three non-mydriatic cameras for diabetic retinopathy screening with artificial intelligence

**DOI:** 10.1038/s41433-024-03000-9

**Published:** 2024-03-11

**Authors:** Mehmet Erkan Doğan, Ahmet Burak Bilgin, Ramazan Sari, Mehmet Bulut, Yusuf Akar, Mustafa Aydemir

**Affiliations:** 1 Department of Ophthalmology, Akdeniz University Faculty of Medicine, Antalya, Turkey; 2 Endocrinology and Metabolic Department, Akdeniz University Faculty of Medicine, Antalya, Turkey; 3 Department of Ophthalmology, Antalya Training and Research Hospital, Antalya, Turkey

**Keywords:** Diabetes complications, Retinal diseases

## Abstract

**Background:**

Diabetic Retinopathy (DR) is a leading cause of blindness worldwide, affecting people with diabetes. The timely diagnosis and treatment of DR are essential in preventing vision loss. Non-mydriatic fundus cameras and artificial intelligence (AI) software have been shown to improve DR screening efficiency. However, few studies have compared the diagnostic performance of different non-mydriatic cameras and AI software.

**Methods:**

This clinical study was conducted at the endocrinology clinic of Akdeniz University with 900 volunteer patients that were previously diagnosed with diabetes but not with diabetic retinopathy. Fundus images of each patient were taken using three non-mydriatic fundus cameras and EyeCheckup AI software was used to diagnose more than mild diabetic retinopathy, vision-threatening diabetic retinopathy, and clinically significant diabetic macular oedema using images from all three cameras. Then patients underwent dilation and 4 wide-field fundus photography. Three retina specialists graded the 4 wide-field fundus images according to the diabetic retinopathy treatment preferred practice patterns of the American Academy of Ophthalmology. The study was pre-registered on clinicaltrials.gov with the ClinicalTrials.gov Identifier: NCT04805541.

**Results:**

The Canon CR2 AF AF camera had a sensitivity and specificity of 95.65% / 95.92% for diagnosing more than mild DR, the Topcon TRC-NW400 had 95.19% / 96.46%, and the Optomed Aurora had 90.48% / 97.21%. For vision threatening diabetic retinopathy, the Canon CR2 AF had a sensitivity and specificity of 96.00% / 96.34%, the Topcon TRC-NW400 had 98.52% / 95.93%, and the Optomed Aurora had 95.12% / 98.82%. For clinically significant diabetic macular oedema, the Canon CR2 AF had a sensitivity and specificity of 95.83% / 96.83%, the Topcon TRC-NW400 had 98.50% / 96.52%, and the Optomed Aurora had 94.93% / 98.95%.

**Conclusion:**

The study demonstrates the potential of using non-mydriatic fundus cameras combined with artificial intelligence software in detecting diabetic retinopathy. Several cameras were tested and, notably, each camera exhibited varying but adequate levels of sensitivity and specificity. The Canon CR2 AF emerged with the highest accuracy in identifying both more than mild diabetic retinopathy and vision-threatening cases, while the Topcon TRC-NW400 excelled in detecting clinically significant diabetic macular oedema. The findings from this study emphasize the importance of considering a non mydriatic camera and artificial intelligence software for diabetic retinopathy screening. However, further research is imperative to explore additional factors influencing the efficiency of diabetic retinopathy screening using AI and non mydriatic cameras such as costs involved and effects of screening using and on an ethnically diverse population.

## Introduction

Diabetic Retinopathy (DR) represents a significant global health concern, contributing substantially to blindness and vision impairment (ref. [1]). DR can be diagnosed through comprehensive retinal examination. The complications associated with proliferative DR and diabetic macular oedema (DMO) can lead to severe vision loss, and in some cases, blindness. However, early diagnosis plays a pivotal role in preventing or effectively treating these complications. Despite the need for regular ophthalmic examinations among diabetic patients to prevent vision loss, less than half of diabetic patients adhere to the recommended examination schedule (ref. [2]).

The TURDEP-II Study, conducted on a large population in Turkey in 2013, found the prevalence of diabetes mellitus (DM) to be 16.5%, corresponding to 6.5 million adults with DM in Turkey (ref. [3]). Compared to the TURDEP-l study performed 12 years earlier, there was a 3% increase in the prevalence of DM (ref. [3]). Currently the prevalence of DM is estimated to be nearly 20% in Turkey. These results, derived from the most extensive nationally representative surveys conducted to date, indicate that DM is one of Turkey’s most complex and widespread public health challenges. Approximately 25-30% of DM patients develop some form of DR, with 3% experiencing a sight-threatening form of the disease (ref. [4]). Unfortunately, in Turkey the insufficient number of ophthalmologists and retina specialists poses a challenge in providing regular retinal screenings for these patients in need.

Certain countries have adopted telemedicine and national screening programs to increase compliance with the retinal examinations required for diagnosing and grading DR (ref. [5]). However, implementing a systematic diabetic retinopathy screening program faces financial and logistical difficulties, particularly in low and middle-income countries like Turkey. Moreover, the sheer size of the population adds complexity and difficulty to introducing a nationwide screening program. Consequently, there is a critical need to develop and validate a cost-effective screening method capable of screening large diabetic populations for DR.

The purpose of DR screening in diabetic patients is to ensure that patients at risk of vision loss can reach the ophthalmologist immediately. Recent studies have highlighted artificial intelligence (AI) algorithms specializing in non-mydriatic posterior pole photographs as a potential solution. Studies on DR diagnosis using AI algorithms have been successfully done in different countries (ref. [6, 7]). Despite the utilization of AI applications in medicine at an academic level in Turkey, no AI algorithm is currently integrated into daily practice for diagnosing and grading DR.

This study evaluated the diagnosis and grading of DR, along with suspicion of clinically significant diabetic macular oedema, by employing the EyeCheckup AI software (EyeCheckup) (Ural Telecommunication Inc., Akdeniz University Teknokent, Antalya), and multiple non-mydriatic ophthalmic cameras. Fundus photographs from patients with diabetes mellitus (DM) were assessed by AI, and these results with those of three retina specialists who established the clinical reference standard using 4 wide-field fundus images.

## Patients and methods

Nine hundred patients with DM diagnosis and without a known DR diagnosis who applied to Akdeniz University Endocrinology and Metabolic Diseases Department were included in the study. Ethics committees of Akdeniz University and the Ministry of Health of the Republic of Turkey approved the study.

The inclusion criteria of patients to participate in the study were as follows:

1. Patients who had DM diagnosis and are followed by the endocrinology department.
2. Older than 18 years of age
3. No previous diagnosis of DR
4. Not having undergone intravitreal injection, laser photocoagulation, or DR-related surgery.
5. Not having undergone an intraocular surgery, including cataract surgery
6. Had signed the informed consent form.
7. The absence of media opacity could affect the retina and optic disc photographic appearance.

**Fig. 1:**
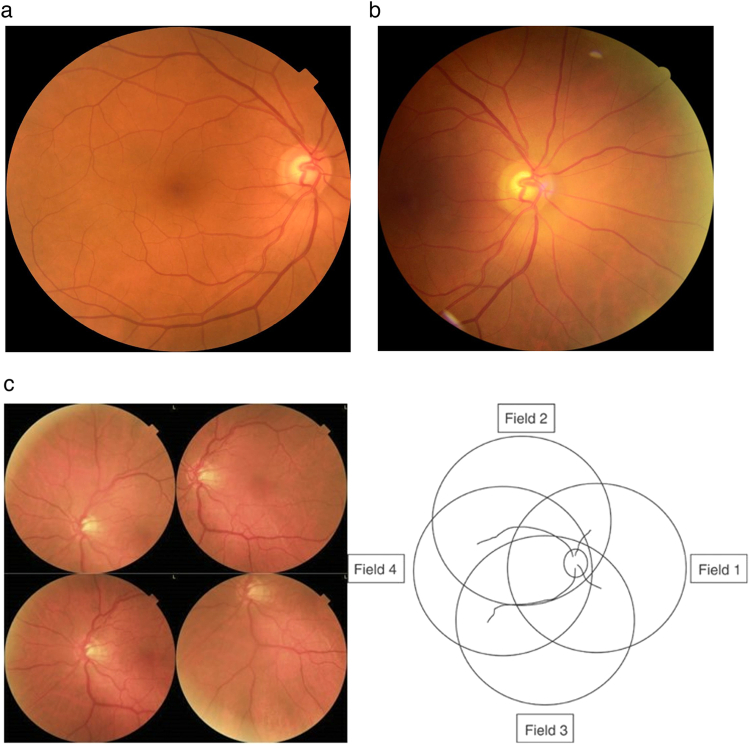
Fundus İmages taken from patients. **a** Non-mydriatic Fundus Images Centered on the Macula from Three Different Cameras. **b** Non-mydriatic Fundus Images Centered on the Optic Disc from Three Different Cameras. **c** 4 Wide Field Imaging.

Posterior pole images were obtained from the patients using three non-mydriatic fundus cameras; Canon CR2 AF, Topcon TRC-NW400, and Optomed Aurora. These images consisted of two wide field images per eye: one centred on the macula (Fig. 1a) and the other centred on the optic disc (Fig. 1b).

**Fig. 2:**
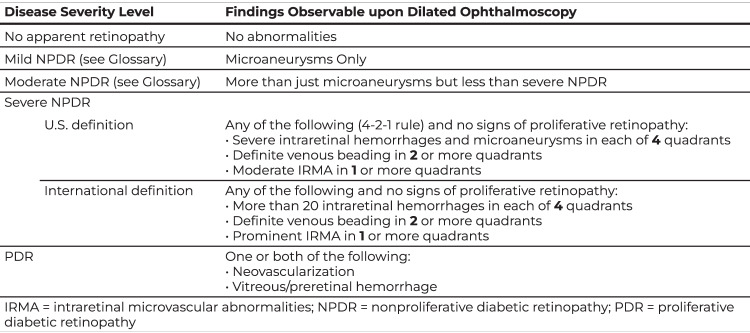
DR severity scale according to american academy of ophthalmology diabetic retinopathy preferred practice pattern.

Two nurses were trained for approximately 4 h prior to the starting date of study on taking two wide field and 4 45 degree wide field fundus images (Fig. 1c) using 3 different cameras according to the camera manufacturer instruction manuals on subjects not included in the study. During the study, the nurses took optic disk-centred and macula-centred Fundus images using each of these cameras without dilation for a maximum of 5 attempts. If images of adequate quality could be obtained without dilation they were analysed using the EyeCheckup software. If the images were of insufficient quality after 5 attempts of non-mydriatic imaging, dilation was performed, and images captured again. Captured images were imported to EyeCheckup client software and evaluated by AI for the presence of DR. EyeCheckup AI software detected the pathological findings (hard exudates, microaneurysms, intraretinal haemorrhages, soft exudates, venous beading, neovascular vessels in the retina and optic disc, preretinal haemorrhage, and vitreous haemorrhage) from the patient’s fundus images. By evaluating the detected pathological findings, these patients were graded as No DR, Mild Non-proliferative DR (NPDR), Moderate NPDR, severe NPDR, and Proliferative DR (PDR), as recommended by the American Academy of Ophthalmology (ref. [8]) (Fig. 2). If the patient had a DR severity greater than mild NPDR, they were graded as “more than mild DR (mtmDR)”. Patients with severe NPDR or PDR were graded as “vision-threatening DR (vtDR)” (ref. [6]). The more than mild diabetic retinopathy group of patients represents the patients that should be referred to an ophthalmologist in 6 months, and the vision threatening diabetic retinopathy group includes those at risk of serious vision loss, thus, should be referred as soon as possible (1–2 months). Historically based on the ETDRS studies (ref. [9]), CSDMO is defined as:

- Retinal thickening at or within 500 μm of the centre of the fovea
- Hard exudates at or within 500 μm of the centre of the fovea if adjacent to an area of retinal thickening

However, since no OCT imaging was involved in this study, the suspicion of CSDMO was determined by surrogate biomarkers near the fovea. Based on the definition of CSDMO in AAO Diabetic Retinopathy Preferred Practice Patterns, the presence of a hard exudate/s in the macula was considered a clinically significant diabetic macular oedema (CSDMO) suspicion(ref. [10]). Patients suspected of clinically significant diabetic macular oedema were also classified as vision threatening diabetic retinopathy.

The subjects underwent dilation and, in addition to the existing two wide field images per eye captured for evaluation by EyeCheckup AI, an additional four quadrant 45 degree wide-field fundus images (Fig. 1c) were also taken from the patients using Canon CR2 AF 45-degree fundus cameras for evaluation by 3 retina specialists. A recent clinical study has shown substantial agreement in the ETDRS 7-Field (7 F) to 4 wide-field (4 W) digital imaging in the evaluation of diabetic retinopathy severity, demonstrating that the two imaging protocols are interchangeable. Both 4 W and 7 F digital imaging protocols can be used for assessing ETDRS levels of DR, even in populations with minimal diabetic retinopathy (ref. [11]). Furthermore, multiple clinical studies show substantial equivalence of using two widefield images compared against 7 standard field images for Diabetic Retinopathy screening using artificial intelligence(refs. [6, 12]).

The four quadrant wide-field images showing the periphery and the previous two non-mydriatic images were evaluated by retina specialists (MED, YA, MB), and a consensus was reached for each eye from each patient. These diagnoses were accepted as ground truth in terms of definitive patient diagnosis. Classification on a patient basis was made by considering the more severe eye. The diagnoses produced by EyeCheckup and the ground truth established by the retinal specialists were compared. During the study the retina specialist and EyeCheckup were blinded to each other’s outputs. Different sensitivity and specificity ratios were calculated for each camera, and the severity of DR was diagnosed. Minimum success thresholds for clinical validation were determined as; 85% for sensitivity and 82% for specificity.

### EyeCheckup AI

EyeCheckup AI has been locally developed at Akdeniz University by the Ural Telecommunication Inc. company that has offered the software for evaluation with multiple cameras. At the time of the study EyeCheckup was the only software explicitly indicated for use with multiple camera models. The IFU for EyeCheckup software according to its CE certification documentation is:

EyeCheckup is indicated for use by healthcare providers to automatically detect mtmDR (more than mild diabetic retinopathy) and vtDR (vision-threatening diabetic retinopathy - severe non-proliferative diabetic retinopathy or proliferative diabetic retinopathy and/or diabetic macular oedema) in the eyes of adults (18 years of age or older) diagnosed with diabetes who have not been previously diagnosed with diabetic retinopathy.

EyeCheckup has been developed for use with retinal fundus images and is optimized with Canon CR-2 AF, Topcon TRC-NW400, and Optomed Aurora fundus cameras. EyeCheckup can visualize the detected anomalies to help doctors understand how diabetic retinopathy detection was made.

### Labelling of the pathologic findings for development of AI

**Fig. 3:**
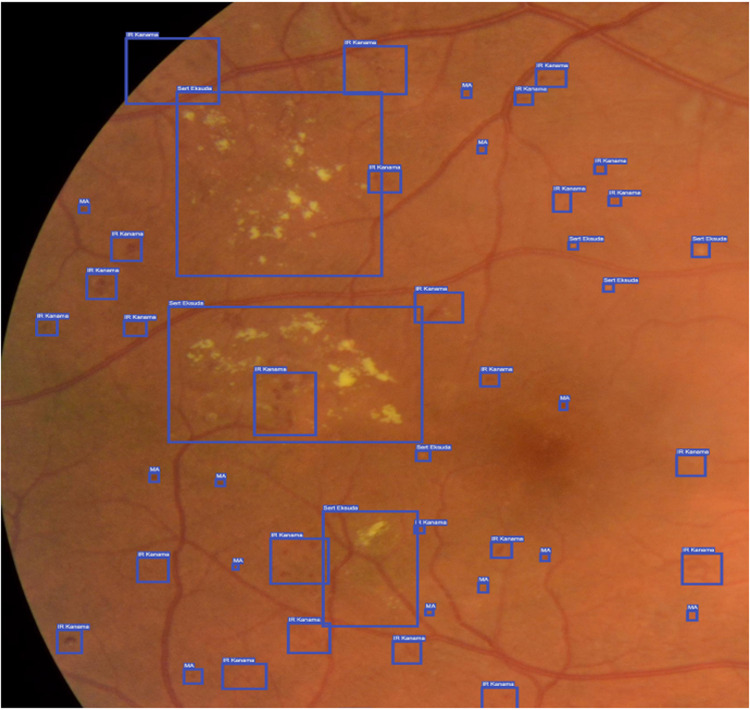
Labelling with doctor says.

For the detection of anomalies in fundus images, an object detection model was developed that was trained on annotated data. Anomalies were labelled using “Doctor Says” labelling software, an open-source tool designed for bounding box labelling, classification, and segmentation (Fig. 3). The labelling process involved identifying specific features in the fundus images that distinguish standard regions from abnormal regions. These features included colour, shape, and texture.

A team of retina experts manually annotated the images using the “Doctor Says” software and the labelling process’s accuracy was verified by cross-validation. The EyeCheckup AI was trained using both public and private images datasets which might contain both dilated and undilated images. These datasets were sorted and labelled by a team of retina specialists in collaboration with machine learning engineers who trained the algorithm. The dataset was enhanced using the undilated images captured using Canon CR2 AF, Topcon TRC-NW400, and Optomed Aurora from the patient population from the Akdeniz University Hospital Ophthalmology Clinic before the study started. A version locked copy of EyeCheckup AI was used in this study and no modification was made anyway including retraining before, in between after the study was completed.

### Preprocessing

Preprocessing is a crucial step in computer vision that involves manipulating, enhancing, and refining raw input data to extract meaningful information effectively. It is a series of techniques and methods designed to optimize the images or videos for subsequent analysis, interpretation, and decision-making. In this context, several quality checks were conducted on the fundus images to ensure they were suitable for analysis.

It is crucial to discuss the importance of preprocessing in computer vision and why it is necessary for accurate and reliable results.

In the preprocessing phase of the study, several quality checks were employed to ensure that the fundus images were suitable for analysis. Firstly, a chromaticity test was implemented to verify whether the images were in colour, discarding those that did not meet this criterion. Secondly, a size threshold check was conducted, retaining only images that met or exceeded 1024 × 1024 pixels. A novel dynamic cropping algorithm was developed to rectify any irregularity in image dimensions that considered the retinal borders and automatically computed an appropriate crop offset. Only images that maintained a post-crop size greater than or equal to 1024 × 1024 pixels were deemed valid for further processing.

Additionally, an optic disc/fovea (ODF) detection model was employed to identify the ODF in each image. By extension, this model provided vital contextual information regarding the image orientation, i.e., whether it pertained to the right or left eye. Potential artifacts that could adversely impact the models’ performance were also addressed, such as partial blurring/darkening, eyelashes in the frame, blurring due to incorrect focus, etc. A quality scoring model was designed and trained to evaluate the images’ suitability for the study to mitigate such issues. Only those images that met this criterion were incorporated into the training and testing datasets.

### EyeCheckup artificial intelligence training process

Approximately 350,000 fundus photographs were collected and evaluated by the quality evaluation model, the anomalies in the picture diagnosed as DR were annotated by the ophthalmologists using only the quality photographs. In the following process, the photos labelled according to the diseases were converted to the appropriate format in line with the models’ needs, and the training of models was trained with the proper architecture. In the training process, architectures were changed, and parameter optimizations were made according to the success of the models; according to the scores obtained from the training, the most successful model was selected.

### EyeCheckup artificial intelligence test process

To comprehensively evaluate the effectiveness of the proposed method, the following metrics were adopted: sensitivity, specificity, and average precision.

True Positive (TP) is the number of positive samples that are correctly identified as positive samples; the number of true negatives (TN) is the number of negative samples that are correctly identified as negative samples, the number of false positives (FP) is the number of negative samples misidentified as positive samples, and the number of false negatives (FN) is the number of positive samples misidentified as negative samples.

The In this study, the primary objective was to determine the DR level of the patient. Converting the predicted anomalies to the DR level is done using the following algorithm using DR disease severity level recommended by the American Academy of Ophthalmology where DR0, DR1, DR2, DR3, and DR4 generated by the software refer to No Apparent Retinopathy, Mild NPDR, Moderate NPDR, Severe NPDR, and PDR respectively in the AAO PPP.

Sensitivity (true positive rate) refers to the probability of a positive test, conditioned on truly being positive. It is calculated as sensitivity = TP/(TP + FN). Specificity (true negative rate) refers to the probability of a negative test, conditioned on truly being negative. It is calculated as specificity = TN/(TN + FP).

### Statistical analysis

The primary endpoints used in this study for the validation of the EyeCheckup AI algorithm are sensitivity and specificity. The sensitivity and specificity values for each camera and disease type were calculated based on the presence or absence of disease as determined by the clinical reference standard and are presented in the following section. Furthermore, a diagnosability statistic was calculated for each camera type. Diagnosability is the ratio of the number of patients whose image quality was sufficient for the EyeCheckup software to make an analysis to the number of patients whose image quality was sufficient for the clinical reference standard to make an evaluation. Lastly, the ratio of patients which required dilation to capture adequate quality images for EyeCheckup usage has also been calculated. These results provide valuable insights into the diagnostic performance of these cameras and their potential use in clinical settings for screening and diagnosis of ocular diseases.

Two-Sided 95% Confidence Intervals are calculated using the Clopper Pearson Exact Binomial method from the RStudio software using the binomial test function.

### Study demographics

900 patients were recruited at the endocrinology department of Akdeniz University Hospital, Antalya, Turkey. Thirty-five of the 900 patients who participated in the study were excluded based on the exclusion criteria. Fundus photographs from 865 patients were included in the study. The study consists of 900 participants, with almost an equal distribution of male (50.89%) and female (49.11%) participants. Most participants have Type 2 diabetes (98.44%), with only a small proportion having Type 1 diabetes (1.56%). On average, participants have been living with diabetes for almost ten years (9.78 years), and the average age of participants is 58 years old. The average weight of participants is 81.52 kilograms, and the average height is 165.28 centimetres, resulting in an average BMI of 29.87. Approximately 3.89% of patients were deemed ineligible for the study, with the remaining 96.11% being eligible for inclusion. Understanding the demographic characteristics of the study population is crucial for interpreting the study results and generalizing them to the larger Population Table 1.

**Table 1:** Study population characteristics for non-mydriatic fundus camera diabetic retinopathy screening trial.

Study population demographics	
Total Study Population	900
Male (%)	458 (50.89%)
Female (%)	442 (49.11%)
Diabetes Type 1 (%)	14 (1.56%)
Diabetes Type 2 (%)	886 (98.44%)
Diabetes Duration in Years (Average)	9.78
Diabetes Duration (Standard Deviation)	8.07
Age in Years (Average)	58.33
Age (Standard Deviation)	11.16
Weight in Kilograms (Average)	81.52
Weight (Standard Deviation)	15.70
Height in Centimetres (Average)	165.28
Height (Standard Deviation)	15.70
BMI (Average)	29.87
BMI (Standard Deviation)	5.37
Ineligible Patients (%)	35 (3.89%)
Eligible Patients (%)	865 (96.11%)

### Determination of sample size

The minimum required sample size was calculated 778, by evaluating whether the AI algorithm achieved a success rate of more than 90%, considering 5% Type-I error, 80% power, and different effect sizes listed below.

**Table:** 

		Given H0	Given H1	Target	Actual		Reject H0
Power	N	(P0)	(P1)	Alpha	Alpha	Beta	If Z > =This
0,8033	5341	0,9000	0,9100	0,0500	0,0511	0,1967	1,6449
0,8002	2286	0,9000	0,9150	0,0500	0,0520	0,1998	1,6449
0,8015	1255	0,9000	0,9200	0,0500	0,0525	0,1985	1,6449
0,8004	778	0,9000	0,9250	0,0500	0,0530	0,1996	1,6449
0,8012	523	0,9000	0,9300	0,0500	0,0541	0,1988	1,6449
0,8047	376	0,9000	0,9350	0,0500	0,0545	0,1953	1,6449
0,8092	284	0,9000	0,9400	0,0500	0,0539	0,1908	1,6449
0,8076	212	0,9000	0,9450	0,0500	0,0565	0,1924	1,6449
0,8004	164	0,9000	0,9500	0,0500	0,0553	0,1996	1,6449

### Statistical results

This study aimed to evaluate the diagnostic accuracy of three different cameras, Optomed Aurora, Canon CR2 AF, and Topcon TRC-NW400, to detect three common ocular anomalies: more than mild diabetic retinopathy, vision threatening diabetic retinopathy, and clinically significant diabetic macular oedema. The Optomed Aurora camera was used for 875 individuals, the Canon CR2 AF camera for 704 individuals, and the Topcon TRC-NW400 camera for 585 individuals. For the EyeCheckupAI software usage a maximum of 526 patients were dilated while being photographed by Topcon NW400 while the other cameras required fewer patients to be dilated, further details regarding dilation statistics can be viewed in Table 2.The Canon CR2 AF camera had a sensitivity and specificity of 95.65% / 95.92% for diagnosing more than mild DR, the Topcon TRC-NW400 had 95.19% / 96.46%, and the Optomed Aurora had 90.48% / 97.21%. For vision threatening diabetic retinopathy, the Canon CR2 AF had a sensitivity and specificity of 96.00% / 96.34%, the Topcon TRC-NW400 had 98.52% / 95.93%, and the Optomed Aurora had 95.12% / 98.82%. For clinically significant diabetic macular oedema, the Canon CR2 AF had a sensitivity and specificity of 95.83% / 96.83%, the Topcon TRC-NW400 had 98.50% / 96.52%, and the Optomed Aurora had 94.93% / 98.95%. The diagnosability of the patients using EyeCheckup software and Optomed, Canon CR2 AF and Topcon TRC-NW400 cameras were 96.57%, 100% and 100% respectively.

**Table 2:** Non-mydriatic fundus cameras for diabetic retinopathy screening: performance metrics.

Optomed aurora (study population = 875)	mtmDR	vtDR	CSDMO
Sensitivity	(114/126)	(78/82)	(75/79)
	90.48%	95.12%	94.93%
	[0.8395–0.9498]	[0.8798–0.9865]	[0.8754–0.9860]
Specificity	(699/719)	(754/763)	(758/766)
	97.21%	98.82%	98.95%
	[0.95737–0.9829]	[0.9777–0.9946]	[0.9795–0.9955]
Accuracy	96.20%	98.40%	98.50%
Diagnosability	845/875		
	96.57%		
Dilation for EyeCheckup Usage	526/845		
	62.24%		
Canon CR2 AF (Study Population = 704)			
Sensitivity	(110/115)	(72/75)	(69/72)
	95.65%	96.00%	95.83%
	[0.9015–0.9857]	[0.8875–0.9917]	[0.8830–0.9913]
Specificity	(565/589)	(606/629)	(612/632)
	95.92%	96.34%	96.83%
	[0.9300–0.9737]	[0.9456–0.9767]	[0.9515–0.9806]
Accuracy	95.80%	96.30%	96.70%
Diagnosability	704/704+		
	100%		
Dilation for EyeCheckup Usage	217/704		
	30.82%		
Topcon NW400 (Study Population = 585)			
Sensitivity	(99/104)	(67/68)	(66/67)
	95.19%	98.52%	98.50%
	[0.8914–0.9842]	[0.9208–0.9996]	[0.9196–0.9996]
Specificity	(464/481)	(496/517)	(499/517)
	96.46%	95.93%	96.52%
	[0.9440–0.9793]	[0.9386–0.9747]	[0.9455–0.9792]
Accuracy	96.20%	96.20%	96.50%
Diagnosability	585/585		
	100%		
Dilation for EyeCheckup Usage	459/585		
	78.46%		

## Discussion

The use case of artificial intelligence in ophthalmology for diabetic retinopathy screening was comprehensively studied and validated during this study. The long-term benefit of AI-based solutions for diabetic retinopathy screening risk stratification and the prognosis is clear (ref. [13]). With the increasing burden of vision loss due to diabetic retinopathy, AI technology can increase the productivity of existing diabetic retinopathy teleophthalmology screening programs managed by trained human graders and ophthalmologists using collaborative semi-automatic models to provide human and machine care (refs. [14, 15]). Since the publication of Abramoff et al.‘s results in 2008, automatic retinal image analysis systems utilizing deep learning algorithms have been developed successfully for detecting diabetic retinopathy based on colour digital retinal images (ref. [7]).

Another comparative study of deep learning versus human graders for classifying diabetic retinopathy severity where the deep learning algorithm had significantly higher sensitivity (0.97 vs. 0.74, *p* < 0.001) and a slightly lower specificity (0.96 vs. 0.98, *p* < 0.001 (ref. [16])). Auto-grading has also been achieved at lower costs when compared to human grading (ref. [17]). Looking at the results of this study which was conducted in a large diabetic population without DR diagnosis, we see that the success thresholds determined before grading of DR and suspicion of clinically significant diabetic macular oedema for the Sensitivity and specificity were significantly exceeded (respectively 85% and 82.5%).

When the more than mild diabetic retinopathy diagnosis success of the EyeCheckup AI algorithm is evaluated with multiple cameras in consideration, the sensitivity recorded was 91% and above; the Specificity was recorded to be 96% and above for more than mild diabetic retinopathy; these diabetic patients require eye care outside of the regular yearly screening. A referral to an ophthalmologist can be made early due to the system’s high sensitivity and specificity.

When evaluating the vision threatening diabetic retinopathy diagnosis accuracy of the EyeCheckup AI software with multiple cameras used, the sensitivity was calculated to be 95% and above, and the specificity was estimated at 96% and above. Vision threatening diabetic retinopathy represents the group of diabetic patients who face the risk of vision loss. The high success achieved in diagnosis suggests that vision loss can be prevented by early referral in some of the patients.

When the patients with suspected clinically significant diabetic macular oedema are evaluated, we see that the sensitivity of the EyeCheckup AI algorithm is 95% and above, and specificity is 96% and above on all cameras. Referral of patients with suspected clinically significant diabetic macular oedema to an ophthalmologist for further examination and treatment may prevent vision loss or allow the loss of sight to return.

When these results are compared with other artificial intelligence studies used to classify diabetic retinopathy, such as Multicenter, Head-to-Head, Real-World Validation Study of Seven Automated Artificial Intelligence Diabetic Retinopathy Screening Systems; the sensitivity ranged from 50.98 to 85.90%, specificity from 60.42 to 83.69%. It has been argued that DR diagnostic algorithms can show significant performance differences (refs. [18, 19]). The breakthrough FDA-approved artificial intelligence study included 900 patients without DRP history in primary care clinics and found a sensitivity of 87.2% and specificity of 90.7% (ref. [6]). When we look at these results, it can be concluded that the AI algorithm studied in this study is far more accurate than any others studied before.

Sensitivity and specificity are critical endpoints in assessing the reliability of an AI algorithm for detecting diabetic retinopathy. While sensitivity ensures that patients at risk of vision loss due to DR are referred to an ophthalmologist for further diagnosis and treatment, specificity aims to minimize the referral of patients who do not have DR or are in the very early stages of the disease. By optimizing both sensitivity and specificity, the unnecessary patient burden on physicians can be reduced, thereby benefiting patients both in terms of time and financial resources. The EyeCheckup AI-assisted diagnosis system has the potential to offer early diagnosis and treatment for diabetic retinopathy in patients who are unable to reach an ophthalmologist. By detecting the disease early on, the system can help prevent vision loss resulting from complications of DR. Moreover, physicians lacking expertise in DR follow-up can utilize the software to determine which patients require further examination and treatment. In this way, the EyeCheckup AI-assisted diagnosis system holds significant promise in improving the overall quality of care for diabetic patients.

This study has several noteworthy strengths. First and foremost, it includes a vast sample size from the intended use. Additionally, various non-mydriatic cameras, including a handheld camera, were used alongside the AI algorithm to diagnose and grade diabetic retinopathy and identify cases of suspected clinically significant diabetic macular oedema (CSDMO). Moreover, the diagnoses generated by the AI were compared to those made by retinal specialists, ensuring a high degree of accuracy and reliability in the study’s findings. This study replicated real-life conditions within an endocrinology clinic. Patients who had not yet been diagnosed with diabetic retinopathy were identified and subsequently referred to the department of ophthalmology for further examination and treatment using an AI solution alongside retina specialists.

A limitation of this study is that suspected cases of clinically significant diabetic macular oedema (CSDMO) were evaluated solely through the retinal fundus. If optical coherence tomography (OCT) had been used, the sensitivity and specificity of the clinically significant diabetic macular oedema diagnosis rather than suspicion could have been assessed. Nonetheless, it is essential to note that the clinically significant diabetic macular oedema suspicion results in this study are primarily based on pathological findings, and a definitive diagnosis cannot be made through photographic evaluation alone. Regardless of this limitation, this study does highlight a valuable outcome for clinics with limited access to OCT technology.

## Conclusion

The EyeCheckup AI model alongside non-mydriatic fundus imaging exhibits adequately high accuracy, sensitivity and specificity in diagnosing diabetic retinopathy and detecting clinically significant diabetic macular oedema (CSDMO) through fundus photographs taken by various non-mydriatic cameras as was demonstrated in this study. The software performed relatively well compared to diagnosis provided by a panel of 3 retina specialists which was used as the ground truth for this study. This robust performance establishes the model as a reliable asset for real-world diabetic retinopathy screening, empowering eye care professionals to deliver more precise and swift diagnoses. Additionally, research indicates that the AI’s analysis of two-field non-mydriatic fundus photographs is comparable to the diagnostic proficiency of retina specialists using dilated four widefield or seven standard field images. These findings highlight the promising potential of AI algorithms in screening diabetic patients who require prompt examination and perhaps treatment by an ophthalmologist, thereby enabling early intervention and improved care.

## Summary

### What was known before

- Differences in diagnostic performance of three non-mydriatic cameras for Diabetic Retinopathy Screening with Artificial Intelligence were unknown

### What this study adds

- It has been found that there are some differences between the diagnostic performance of the three non-mydriatic cameras for Diabetic Retinopathy Screening with Artificial Intelligence

## Data Availability

Data is available from the authors upon reasonable request.
